# Rapid Assessment of Distribution of Wildlife and Human Activities for Prioritizing Conservation Actions in a Patagonian Landscape

**DOI:** 10.1371/journal.pone.0127265

**Published:** 2015-06-10

**Authors:** Lorena F. Rivas, Andrés J. Novaro, Martín C. Funes, R. Susan Walker

**Affiliations:** 1 Instituto de Investigaciones en Biodiversidad y Medioambiente, Consejo Nacional de Investigaciones Científicas y Técnicas, Junín de los Andes, Neuquén, Argentina; 2 Patagonian Steppe Program, Wildlife Conservation Society, Junín de los Andes, Neuquén, Argentina; Università degli Studi di Napoli Federico II, ITALY

## Abstract

Large landscapes encompassing reserves and areas with other human uses are necessary for conservation of many species. Generating information for conservation planning over such landscapes may be expensive and time-consuming, though resources for conservation are generally limited and conservation is often urgent. We developed a sign-based occupancy survey to help prioritize conservation interventions by simultaneously assessing the distribution of 3 species, the lesser rhea, guanaco, and mara, and their association with human activities in a 20,000-km^2^ landscape in the northern Patagonian steppe. We used a single-season occupancy model with spatial rather than temporal replication of surveys in order to reduce costs of multiple visits to sites. We used covariates related to detectability, environmental factors, and different human activities to identify the most plausible models of occupancy, and calculated importance weights of covariates from these models to evaluate relative impacts of human activities on each species. Abundance of goats had the strongest negative association with lesser rheas and guanacos, and road density with maras. With six months of fieldwork, our results provided initial hypotheses for adaptive conservation interventions for each species. Addressing high livestock densities for rheas and guanacos, poaching by urban hunters for all three species, and hunting by rural people for rheas are priorities for conservation in this landscape. Our methodology provided new insights into the responses of these species, although low detection probabilities for maras indicate that the sampling scheme should be altered for future monitoring of this species. This method may be adapted for any large landscape where a rapid, objective means for prioritizing conservation actions on multiple species is needed and data are scarce.

## Introduction

Conservation only within protected areas is insufficient in most parts of the world, especially for large-bodied, wide-ranging species. To plan conservation at a meaningful scale for these species, large landscapes that include protected areas as well as areas with other human uses must be considered [1], [2]. Conservation action at this scale is complex due to multiple jurisdictions and stakeholders. Collection of data on target species and human activities in order to prioritize and guide conservation actions may be expensive and time-consuming. Nevertheless, action at this scale is often necessary to improve long-term prospects for persistence of these species [3]

Surveys of a random set of sites for presence of a species may be used for rapid evaluation of distribution of the species within a large landscape. However, failure to account for the probability of a species being present without being detected leads to underestimation of distribution and thus biases conclusions [4]. Recent developments in occupancy modeling provide means for estimating the detection probability and reducing bias through multiple sampling. This methodology has been used increasingly for a variety of purposes at a landscape scale, such as prioritizing conservation planning for forest birds [5], analyzing effects of land use on carnivore diversity [6], evaluating effectiveness of a regional, multi-species conservation plan [7], and determining the landscape-level distribution of tigers [8].

The Patagonian steppe and scrub of southern Argentina, like most habitats worldwide, has been drastically changed over the last century by human activities. Only 0.7% is designated as protected areas with management plans and regular ranger patrols [9]. Conservation of wildlife in this region requires both strengthening of protected areas and improving conditions for wildlife outside of protected areas. Our target species for conservation in a 20,000-km^2^ landscape in the northern Patagonian steppe include the guanaco *Lama guanicoe*, lesser rhea *Rhea pennata*, and mara *Dolichotis patagonum*.

Our overall goal was to develop a conservation strategy for these species in the landscape, and to that end we sought to identify how addressing different potential threats might impact their status. The principal objective of the study reported here was to quickly evaluate the distribution of lesser rheas and the human activities associated with them in the large, multi-use landscape. The lesser rhea, a 15–25 kg flightless bird [10] has declined throughout its range due to habitat degradation as a result of overgrazing, competition with livestock, predation, collection of eggs, and illegal hunting [11–14]. Within the landscape, we had data on distribution of the species from only one small sector. We needed to rapidly assess its overall distribution and simultaneously evaluate which threats were most relevant to address in this landscape. Thus, the study was designed with the lesser rhea in mind, a species that historically probably occupied most of the landscape except for the highest altitudes.

Our secondary objective was to evaluate distributions and human activities associated with the distributions of our other target species in this landscape, the mara and the guanaco. The mara is a large (8–16 kg) burrowing rodent restricted to areas with adequate soils. It is considered to be declining, due to loss of habitat, competition with livestock and introduced lagomorphs, and hunting [15]. The current distribution of the mara within the landscape was not known, but we do expect that historically it was limited to areas with adequate habitat conditions. The guanaco, a South American camelid, is the dominant herbivore of the steppe and scrub, and has suffered a 60% reduction of its range due to hunting, competition with livestock, and habitat loss [16]. This species also probably historically used the entire landscape. We had more detailed knowledge of its distribution and abundance within the landscape than for the other species, as well as information on human activities affecting its abundance at specific sites [17]. Nevertheless, we sought to evaluate human activities associated with the distribution of this species throughout the landscape, in order to evaluate potential landscape-level impacts of addressing those activities.

The human activities we considered to potentially have the greatest impacts on wildlife distribution and abundance were hunting and livestock husbandry. There are two principal types of hunting in the region, hunting by poachers, mostly urban residents, from vehicles along roads, and hunting by rural people from horseback. Interventions for the two types of hunting would be different. Livestock husbandry may affect native herbivores through various mechanisms, including direct competition for food and water, persecution by livestock producers, disease transmission from livestock to wildlife, and apparent competition between livestock and native prey via supplementation of native predators by livestock [13], [17–24].

Our design, based on occupancy modeling with covariates related to different human activities, permitted us to simultaneously evaluate distribution of these species and collect preliminary information on the most relevant human activities to target for adaptive management over the landscape. This approach could be useful to plan conservation for large, mixed-use areas where information on distribution of target species and human activities, as well as relationships between animal distributions and human activities, is needed in a short time period.

## Materials and Methods

### Study area

**Fig 1:**
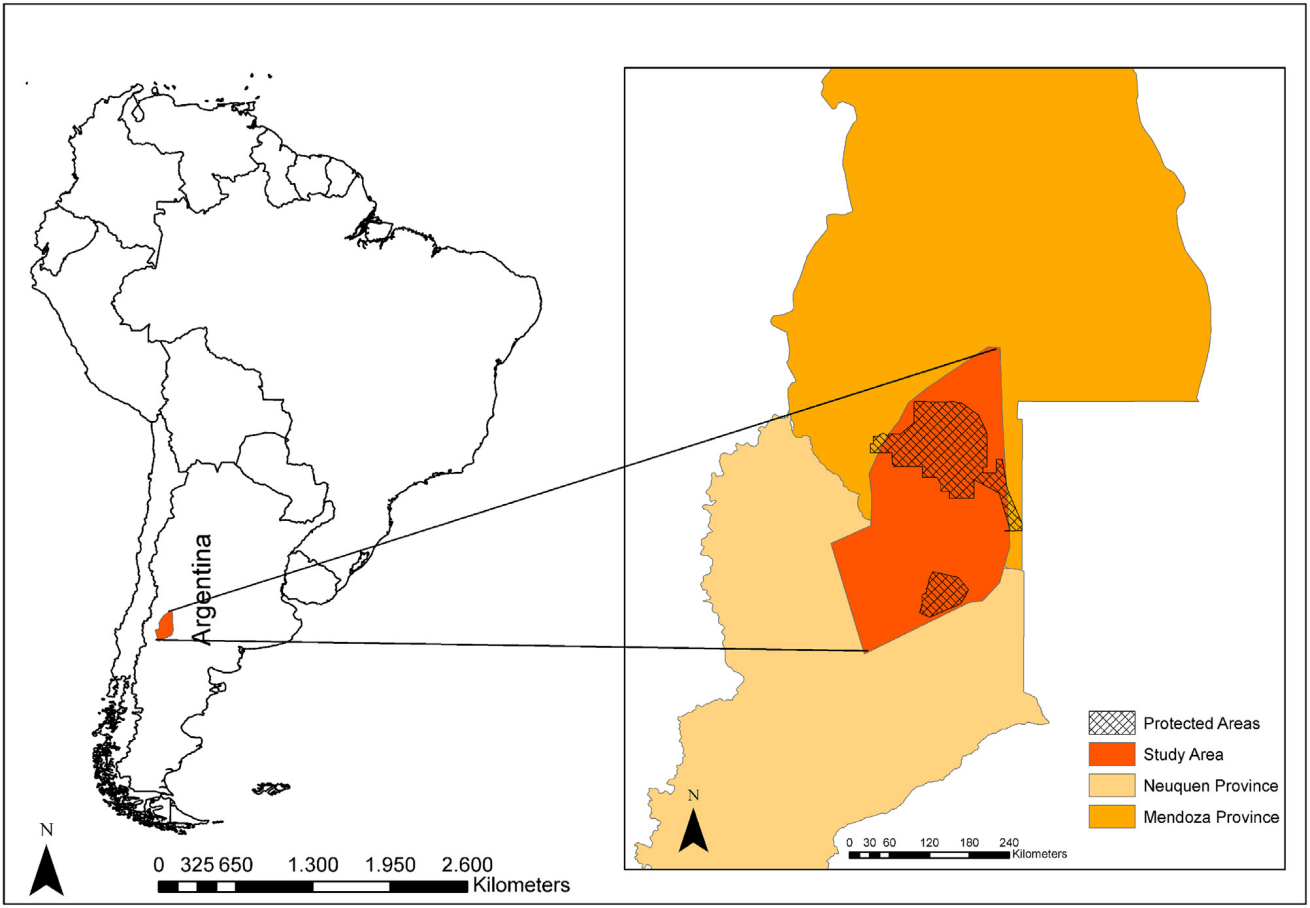
Study area situated in South America.

The 20,000-km^2^ study area in northern Patagonia (Fig 1) is a mosaic of three biomes: the Patagonian steppe, scrub, and high Andes. The topography consists of high plateaus, river valleys, and old volcanic cones up to 4700 m.a.s.l. in elevation. Due to this geography and topography, the region is one of the areas of highest biodiversity of arid Patagonia [9]. It encompasses two large reserves (Reserva Provincial Auca Mahuida, Neuquén province, 770 km^2^, and Reserva La Payunia, Mendoza province, 6000 km^2^) and overlaps with the most productive oil field in Argentina. The predominant activity of local people is small-scale goat husbandry.

### Data collection

We divided the study area into a grid of 2 km by 2 km cells, and eliminated those that were inaccessible due to altitude and lack of trails or roads. We used the program PRESENCE (http://www.mbr-pwrc.usgs.gov/software) to determine the sampling scheme based on simulations using detection and occupancy probabilities from a pilot study on lesser rheas with similar survey methodology. We determined that with 105 sites sampled 2 times, including a subset of 20 sites sampled more intensively (4 times), we could achieve an occupancy estimate of 0.826, S.E. = 0.032, when true occupancy was 83%. We randomly selected 105 cells to sample from the 4741 cells in the grid. We used a single-season design, and because of the high cost in time and money of reaching each site, we did repeated spatial sampling of each site on the same day, rather than repeating sampling over time [25]. Sampling was carried out from September 2008 to March 2009, during the austral spring and summer.

We sampled signs rather than using direct observation of animals on transects due to relative low density of all species, which would lead to low sample sizes. The pilot study and other previous work by our group on lesser rheas demonstrated that sign transects and transects based on direct sightings of rheas are highly correlated, and that use of direct sightings tends to underestimate rhea abundance, especially in areas of low density [26]. Sampling was based on observation of signs while walking along 1000-m transects. The cells to be sampled more intensively and the point of origin and orientation of each transect were chosen randomly.

We recorded feces and other signs (carcasses, tracks, feathers) of lesser rheas, guanacos, and maras encountered along each transect. We recorded the age category of rhea feces as either “fresh” (green inside and outside), semi-fresh (green inside and grey outside), and old (grey outside and partially disintegrated). A transect was considered to be occupied by the species if at least one sign of that species was found in the transect. In addition, to estimate the proportion of each transect used by livestock and exotic species, we placed a 1-m diameter ring on the ground every 10 meters of each transect and recorded whether signs of sheep, goats, cows, horses, European hares, and/or European rabbits were found within the ring. The proportion of the transect occupied by large (cows/horses) and small (sheep/goats) livestock and exotic lagomorphs was calculated as the number of rings with feces of these species divided by 100 (number of rings).

For each cell we estimated covariates related to habitat, hunting, and livestock husbandry (Table 1). Habitat covariates included NDVI, as a proxy for productivity, elevation, and slope. NDVI is the Normalized Difference Vegetation Index, a simple graphical indicator that assesses the coverage of live green vegetation. Working with 0.05° images with monthly NDVI from the IDRISI 16 archive derived from 0.5 × 0.5°MODIS satellite images processed by the NASA Goddard Space Flight Center [27], we extracted the average NDVI for each cell for the month in which it was sampled. The elevation of a cell was calculated as the average elevation of the starting and ending points of the cell’s transects, estimated with a hand-held GPS. Slope was calculated as the average percent of slope per cell, obtained via the digital elevation model (DEM) using Spatial Analyst in ArcMap 9.2 (ESRI) [28].

**Table 1:** Occupancy covariates and range of values.

	Variable (Acronym)	Range (units)
Habitat	NDVI	1152–2531
	Percent slope (SLOPE)	0.35–13.67
	Elevation (ELEV)	348.5–1951 (m.a.s.l.)
Hunting	Road density (DENSROAD)	0–14.46 (km/km^2^)
	Distance to the nearest road (DISTROAD)	0.006–26.22 (km)
	Distance to nearest ruralresidence (DISTRES)	0.27–45.89 (km)
	Distance to the nearest town (DISTOWN)	4.14–55.93 (km)
Livestock	Proportion of transect with signs of cows/horses (COW/HORSE)	0–0.71
	Proportion of transect with signs of goats/sheep (GOAT/SHEEP)	0–0.86
Introduced species	Proportion of transect with signs of hares/rabbits (HARE/RABBIT)	0–0.51

We used density of roads within a cell (including oil exploration trails), distance to the nearest public road, and distance to the nearest town as measures of access by poachers [17], and distance to the nearest rural residence as a measure of pressure of hunting by rural people (Table 1). For livestock husbandry, we used the proportion of each transect used by large and small livestock, as described above. For maras we also included introduced lagomorphs as potential competitors. We digitized roads and passable oil exploration trails as seen in Google Earth 6.0 and calculated the density (km/km^2^) of these roads within each cell sampled using ArcGIS 9.2 (ESRI, Redlands, California, USA). Locations of nearest rural residences were recorded in the field with a GPS or visualized in Google Earth and added as a GIS point layer. We measured the distance from the central point of each cell to the nearest town, nearest road, and nearest rural residence in ArcGIS.

Finally, we also considered factors that could affect the detection of signs in the transects and included these as covariates for the estimation of the probability of detection. Because the signs are on the ground in areas of mostly low coverage and stature of vegetation, we considered that the angle of the sun, as reflected by the time of day (morning/midday/afternoon) could affect our ability to see signs. Most transects were carried out by the first author, so we also considered her greater experience with detection of signs to be a possible influence on detection probability and included the observer as an additional covariate in the estimation of detection probability.

We received permission for surveys in the Auca Mahuida Reserve from the Dirección Provincial de Recursos Faunísticos y Áreas Protegidas de Neuquen and for surveys in La Payunia Reserve from the Dirección de Recursos Naturales Renovables de Mendoza. Remaining surveys were on private lands where we obtained permission from the owners to conduct surveys at each site. Approval by an Institutional Animal Care and Use Committee (IACUC) was not required because the study was based on observation of signs, and we did not handle any animals.

### Data analysis

We used occupancy models calculated with the program PRESENCE to evaluate the probability of occupancy by different species [4], [29]. These models are based on a maximum likelihood method to estimate occupancy when the probability of detection of a species is < 1. Occupancy models provide an estimation of occupancy (*psi*) that incorporates the probability of detection (*p*). Inclusion of covariates for occupancy provides a means for evaluating their impact on occupancy, and covariates for detection provide more robust estimations of occupancy. We first modeled detection probability with covariates, and for each species identified those that improved the models relative to models without the covariates. For each species we used the detection covariates in the model with the lowest Akaike’s Information Criteria (AIC) in subsequent modeling of occupancy.

We calculated Pearson’s correlations between all covariates for occupancy, and then evaluated models with all combinations of uncorrelated variables (Table 2). Variables with wide ranges of values that were not close to 1 (NDVI, slope, elevation, road density, distance to nearest rural residence, distance to nearest town) were normalized. We evaluated goodness of fit for the most complete models using the Mackenzie-Bailey adaptation of the Pearson’s chi square test [30].

**Table 2:** Correlations between covariates.

	NDVI	Densroad	Slope	Elev	Distres	Distown	Distroad	Cow/ horse	Goat/ sheep	Hare/ rabbit
NDVI	1.00	**-0.28**	**0.32**	**0.45**	0.04	-0.08	-0.11	-0.11	**0.23**	0.04
Densroad	**-0.28**	1.00	**0.20**	**-0.31**	0.01	-0.14	-0.08	-0.02	-0.11	-0.03
Slope	**0.32**	**0.20**	1.00	0.17	-0.17	-0.02	-0.13	**-0.21**	0.16	0.10
Elev	**0.45**	**-0.31**	0.17	1.00	**0.24**	-0.04	**-0.22**	-0.03	0.01	**0.44**
Distres	0.04	0.01	-0.17	**0.24**	1.00	0.14	-0.01	-0.08	**-0.28**	0.16
Distown	-0.08	-0.14	-0.02	-0.04	0.14	1.00	**0.41**	0.13	0.07	0.04
Distroad	-0.11	-0.08	-0.13	**-0.22**	-0.01	**0.41**	1.00	0.06	0.00	-0.06
Cow/horse	-0.11	-0.02	**-0.21**	-0.03	-0.08	0.13	0.06	1.00	0.04	**0.38**
Goat/sheep	**0.23**	-0.11	0.16	0.01	**-0.28**	0.07	0.00	0.04	1.00	-0.06
Hare/rabbit	0.04	-0.03	0.10	**0.44**	0.16	0.04	-0.06	**0.38**	-0.06	1.00

Marked correlations are significant at p < 0.005. N = 105. Densroad = road densityElev = elevation,Distres = distance to the nearest residence, Distown = distance to the nearest town, Distroad = distance to the nearest road, Cow-horse = proportion of transect with signs of cows or horses, Goat-sheep = proportion of transect with signs of goats or sheep, Hare/rabbit = proportion of transect with signs of hares or rabbits.

For each occupancy model we calculated the AIC, which provides a measure of fit and precision of the model, and ordered models from lowest to highest AIC. Unlike in traditional statistical analyses, we did not seek a single “best” model, but rather searched for models that improved the estimate of occupancy and the covariates associated with those models [31]. For each model we calculated the normalized Akaike weight as a measure of relative plausibility. We calculated the change in Akaike weight between each model and the model with the lowest AIC (delta AIC) and considered that a delta AIC <2 indicated that a model was equally plausible as the model with the lowest AIC. To assess the relative importance of each covariate in relation to the presence of the species we calculated an importance weight for all covariates in models with delta AIC <2 by summing the AIC weight of each model containing that variable [32]. This allowed us to make inferences about the relative importance of individual covariates when several models were supported nearly equally.

As models with covariates provide site-specific estimates of occupancy and detectability, we report the range of probabilities of occupancy and standard errors of occupancy for different sites under each model. To provide a measure of variability for these, we weighted each site-specific occupancy and standard error estimate by the model weight and summed over all of the equally plausible models. We averaged these and calculated an overall weighted coefficient of variation (CV) by dividing the weighted standard errors by the weighted *psi* for each site and averaging over all sites. Finally, to get an overall estimate of potential impact of addressing different threats on the suite of species, we summed the importance weights for human-related covariates across all three species [6].

## Results

**Fig 2:**
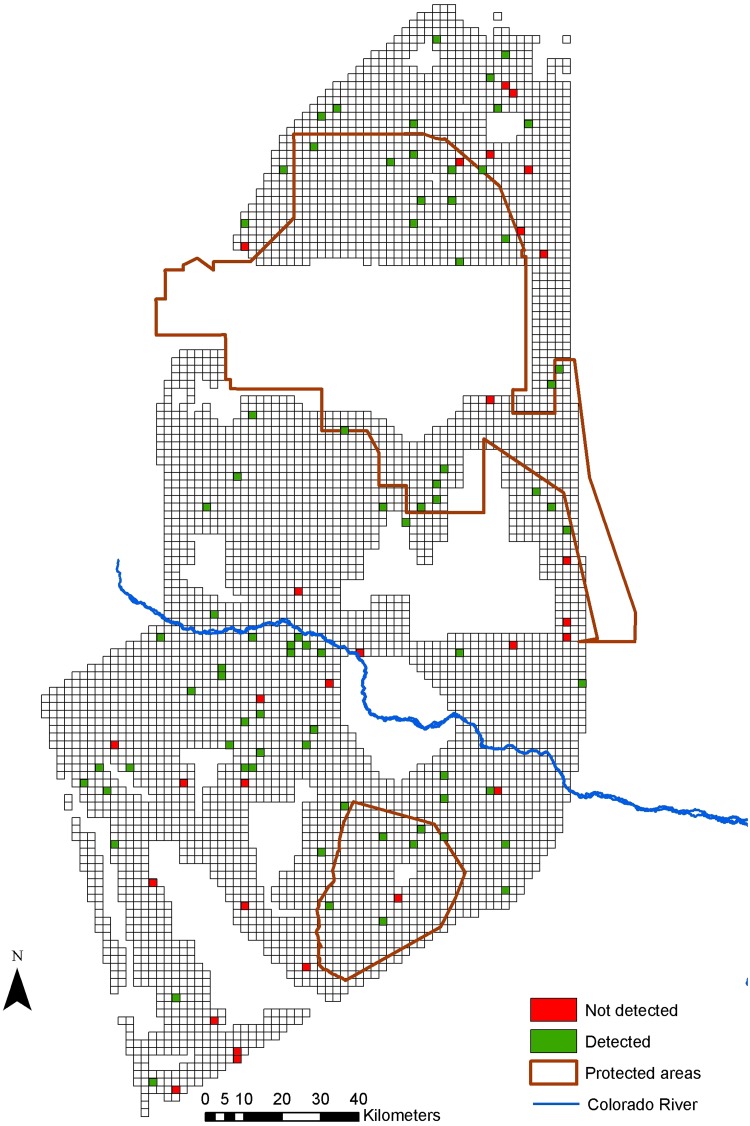
Landscape divided into a grid with sampled cells where lesser rheas were detected or were not detected.

**Fig 3:**
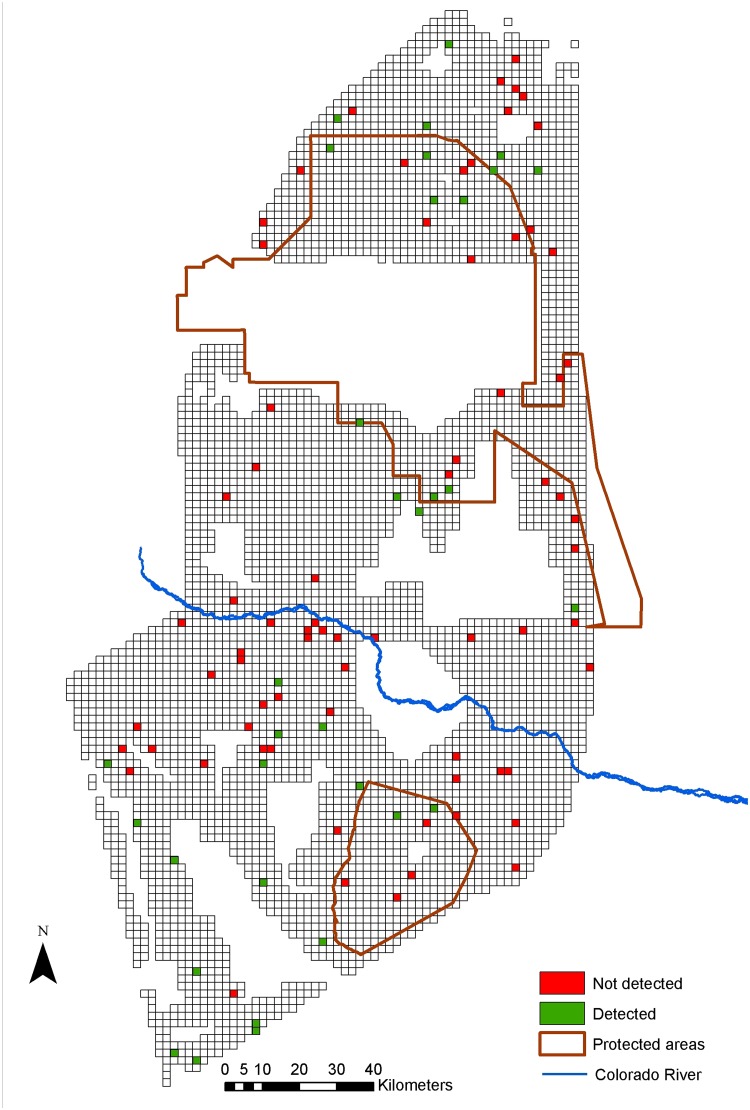
Landscape divided into a grid with sampled cells where maras were detected or were not detected.

**Fig 4:**
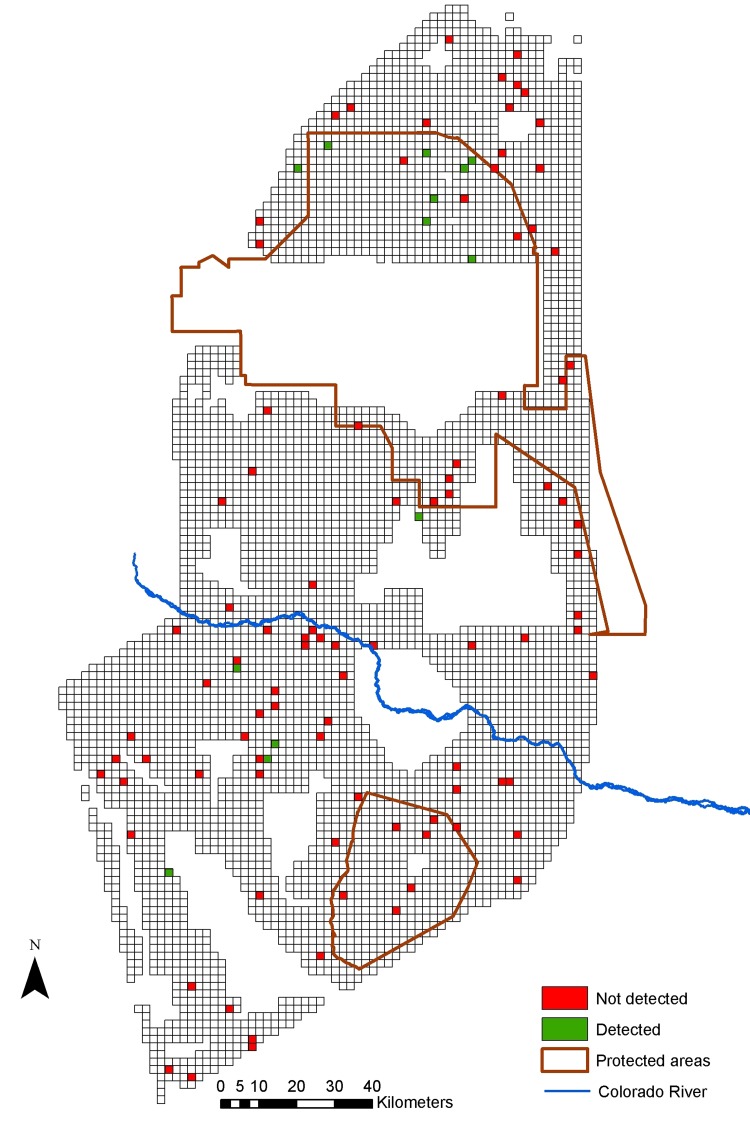
Landscape divided into a grid with sampled cells where guanacos were detected or were not detected.

We found evidence of lesser rheas in 72% (naïve occupancy estimate) of the cells, of maras in 31%, and of guanacos in 12% (Figs 2–4). Lesser rheas were found throughout most of the study area, and maras were absent from the northernmost part of the study area and around the Rio Colorado. Most guanaco signs were encountered within the Payunia Reserve, which protects the region’s largest population. For lesser rheas, the most complete occupancy model using only fresh and semi-fresh feces did not fit the data (χ^2^ = 49.267, p = 0.0099, df = 7), but the model including all feces regardless of their age category did (χ^2^ = 26,474 p = 0.277, df = 7). Therefore, we used feces of all age categories in the remainder of the analyses. The most complete models for mara (χ^2^ = 16.162, p = 0.505, df = 5) and guanaco (χ^2^ = 28.521, p = 0.178, df = 6) fit the data.

In the detection probability models for lesser rheas, the lowest AIC was obtained using the time of day as a covariate (Table 3). Detectability of lesser rhea signs was very high, with estimates ranging from 0.739 to 0.904 when time of day was included as a covariate (Table 4). Probability of detection was greatest at midday, lower in the morning, and lowest in the afternoon. Ten models were equally plausible (delta AIC < 2; Table 4). Lesser rheas were found in higher, steeper areas, with lower productivity, though elevation and slope were of relatively low importance. With respect to livestock, lesser rheas were more likely to be found where there were fewer sheep, goats, cows, and horses, with sheep and goats having a much greater importance than large livestock. Finally, for the covariates related to hunting, lesser rheas were more likely to be found closer to roads and farther from rural residences, in areas with fewer roads (Tables 5 and 6). Three covariates had much greater importance weights than the others: goat/sheep (-), distance to nearest road (-), and NDVI (-) (Table 5). Probability of occupancy of different sites (psi) under the models ranged from 0.31 to 0.93, with a weighted average CV of 10% (Table 4).

**Table 3:** AICs of models with detection and without detection covariates.

SPECIES	No covariates	Time	Observer
Lesser rhea	291.46	**290.02**	292.97
Mara	**226.83**	229.97	228.25
Guanaco	177.48	181.07	**174.49**

**Table 4:** Lesser rhea model with lowest AIC and all models within a delta AIC of <2.

							PSI				P			
							Estimate		SE		Estimate		SE	
MODEL	AIC	Delta AIC	AIC weight	Model Likelihood	Number of parameters	-2. LogLikelihood	MIN	MAX	MIN	MAX	MIN	MAX	MIN	MAX
psi(ndvi,distroad),p(time)	284.89	0	0.114	1	6	272.89	0.323	0.923	0.044	0.259	0.739	0.904	0.046	0.055
psi(goat-sheep),p(time)	284.9	0.01	0.113	0.995	5	274.9	0.354	0.84	0.048	0.153	0.741	0.902	0.047	0.055
psi(ndvi),p(time)	285.14	0.25	0.100	0.883	5	275.14	0.360	0.910	0.048	0.155	0.742	0.904	0.046	0.055
psi(distroad,goat-sheep),p(time)	285.51	0.62	0.083	0.733	6	273.51	0.345	0.870	0.049	0.261	0.738	0.903	0.047	0.055
psi(densroad,goat-sheep),p(time)	286.19	1.3	0.059	0.522	6	274.19	0.351	0.870	0.048	0.212	0.741	0.901	0.048	0.055
psi(densroad,distroad,goat-sheep),p(time)	286.62	1.73	0.048	0.421	7	272.62	0.346	0.894	0.049	0.222	0.739	0.903	0.047	0.055
psi(elev,goat-sheep),p(time)	286.7	1.81	0.046	0.405	6	274.7	0.350	0.866	0.048	0.159	0.741	0.901	0.048	0.055
psi(ndvi,distroad,cow-horse),p(time)	286.72	1.83	0.046	0.401	7	272.72	0.332	0.932	0.046	0.260	0.739	0.905	0.046	0.055
psi(slope,goat-sheep),p(time)	286.78	1.89	0.044	0.389	6	274.78	0.313	0.922	0.045	0.269	0.740	0.904	0.047	0.055
psi(ndvi,distres,distroad),p(time)	286.78	1.89	0.044	0.389	7	272.78	0.364	0.874	0.048	0.179	0.741	0.902	0.047	0.055

Minimum (MIN) and maximum (MAX) occupancy (PSI) and detectability (P) estimates and minimum and maximum standard errors for Psi and P for individual sites for each model. elev = elevation,densroad = road density, distres = distance to the nearest residence, distroad = distance to the nearest road, cow-horse = proportion of transect with signs of cows or horses, goat-sheep = proportion of transect with signs of goats or sheep, time = whether transect was done in the morning, around noon, or in the afternoon.

**Table 5:** Importance weight and direction of relationship of each covariate for lesser rhea occupancy.

VARIABLE	IMPORTANCE WEIGHT	RELATIONSHIP
Goat-Sheep	0.394	(-)
Distance to the nearest road	0.335	(-)
NDVI	0.304	(-)
Road density	0.107	(-)
Elevation	0.046	(+)
Cow-Horse	0.046	(-)
Slope	0.044	(+)
Distance to the nearest residence	0.044	(+)

**Table 6:** Beta estimates and standard errors for covariates included in all equally plausible models for lesser rhea occupancy.

	PSI (OCCUPANCY)									P (DETECTABILITY)	
MODEL		Goat-Sheep	Distroad	NDVI	Densroad	Elev	Cow-Horse	Slope	Distres	Time2	Time3
psi(ndvi,distroad),p(time)	beta	X	-0.364	-0.727	X	X	X	X	X	0.359	-0.847
	SE	X	0.234	0.277	X	X	X	X	X	0.713	0.549
psi(goat-sheep),p(time)	beta	-2.618	X	X	X	X	X	X	X	0.335	-0.831
	SE	0.993	X	X	X	X	X	X	X	0.709	0.547
psi(ndvi),p(time)	beta	X	X	-0.644	X	X	X	X	X	0.355	-0.829
	SE	X	X	0.256	X	X	X	X	X	0.712	0.548
psi(distroad,goat-sheep),p(time)	beta	-2.690	-0.284	X	X	X	X	X	X	0.350	-0.843
	SE	1.009	0.229	X	X	X	X	X	X	0.708	0.547
psi(densroad,goat-sheep),p(time)	beta	-2.721	X	X	-0.233	X	X	X	X	0.327	-0.832
	SE	1.010	X	X	0.265	X	X	X	X	0.711	0.547
psi(densroad,distroad,goat-sheep),p(time)	beta	-2.804	-0.309	X	-0.275	X	X	X	X	0.344	-0.843
	SE	1.025	0.234	X	0.278	X	X	X	X	0.712	0.547
psi(elev,goat-sheep),p(time)	beta	-2.601	X	X	X	0.127	X	X	X	0.330	-0.830
	SE	0.989	X	X	X	0.283	X	X	X	0.711	0.547
psi(ndvi,distroad,cow-horse),p(time)	beta	X	-0.361	-0.739	X	X	-0.713	X	X	0.364	-0.844
	SE	X	0.233	0.279	X	X	1.721	X	X	0.712	0.549
psi(slope,goat-sheep),p(time)	beta	-2.687	X	X	X	X	X	0.096	X	0.341	-0.827
	SE	1.018	X	X	X	X	X	0.275	X	0.708	0.547
psi(ndvi,distres,distroad),p(time)	beta	X	-0.359	-0.723	X	X	X	X	0.086	0.354	-0.842
	SE	X	0.233	0.275	X	X	X	X	0.261	0.714	0.548

elev = elevation,densroad = road density, distres = distance to the nearest residence, distroad = distance to the nearest road, cow-horse = proportion of transect with signs of cows or horses, goat-sheep = proportion of transect with signs of goats or sheep, time2 = transect done around noon, time3 = transect done in the afternoon (relative to transects done in the morning).

For maras, neither time of day nor observer as detection probability covariates lowered the AIC (Table 3). Detection probability was very low (mean = 0.13) and imprecise (mean S.E. = 0.30; Table 7). Thirteen models were equally plausible (Table 7). In terms of habitat, maras were found in flatter areas of higher productivity. Maras were positively associated with livestock and exotic lagomorphs. With respect to covariates related to hunting, maras were more likely to be present in areas with fewer roads, and closer to roads and residences (Tables 8 and 9). Road density was the covariate with the greatest importance weight for maras, with a weight two times greater than that of the covariate with the next highest weight (Table 8). Probability of occupancy per site under different models ranged from 0.06 to 0.78, with an overall weighted average CV of 26% (Table 7).

**Table 7:** Mara model with lowest AIC and all models within a delta AIC of <2.

							PSI				P	
							Estimate		SE			
MODEL	AIC	Delta AIC	AIC weight	Model Likelihood	Number of parameters	-2.Log Likelihood	MIN	MAX	MIN	MAX	Estimate	SE
psi(densroad),p(.)	224.53	0	0.073	1	3	218.53	0.071	0.498	0.063	0.092	0.149	0.292
psi(densroad,cow-horse),p(.)	224.83	0.3	0.063	0.861	4	216.83	0.077	0.631	0.067	0.208	0.129	0.296
psi(densroad,goat-sheep),p(.)	224.83	0.3	0.063	0.861	4	216.83	0.057	0.692	0.065	0.195	0.144	0.294
psi(densroad,distres),p(.)	225.09	0.56	0.05	0.756	4	217.09	0.056	0.578	0.066	0.144	0.145	0.292
psi(densroad,cow-horse,goat-sheep),p(.)	225.26	0.73	0.051	0.694	5	215.26	0.062	0.662	0.071	0.204	0.129	0.296
psi(slope,goat-sheep,hr),p(.)	225.81	1.28	0.038	0.527	5	215.81	0.105	0.744	0.066	0.279	0.163	0.291
psi(densroad,distroad),p(.)	226.15	1.62	0.032	0.445	4	218.15	0.076	0.52	0.065	0.216	0.144	0.293
psi(hr),p(.)	226.4	1.87	0.029	0.393	3	220.4	0.279	0.733	0.064	0.217	0.112	0.301
psi(densroad,distroad,goat-sheep),p(.)	226.42	1.89	0.028	0.389	5	216.42	0.062	0.688	0.069	0.221	0.137	0.295
psi(distres,hr),p(.)	226.45	1.92	0.028	0.383	4	218.45	0.105	0.620	0.064	0.221	0.111	0.299
psi(ndvi,hr),p(.)	226.46	1.93	0.028	0.381	4	218.46	0.199	0.71	0.065	0.224	0.118	0.299
psi(ndvi,cow-horse),p(.)	226.47	1.94	0.028	0.379	4	218.47	0.171	0.690	0.064	0.183	0.123	0.297
psi(ndvi,distres,hr),p(.)	226.5	1.97	0.027	0.373	5	216.5	0.126	0.780	0.067	0.211	0.125	0.295

Minimum (MIN) and maximum (MAX) occupancy (PSI) and detectability (P) estimates and minimum and maximum standard errors for Psi and P for individual sites for each model. elev = elevation,densroad = road density, distres = distance to the nearest residence, distroad = distance to the nearest road, cow-horse = proportion of transect with signs of cows or horses, goat-sheep = proportion of transect with signs of goats or sheep, hr = proportion of transect with signs of introduced rabbits and/or hares.

**Table 8:** Importance weight and direction of relationship of each covariate for mara occupancy.

VARIABLE	IMPORTANCE WEIGHT	RELATIONSHIP
Road density	0.365	(-)
Cow-Horse	0.141	(+)
Goat-Sheep	0.180	(+)
Distance to the nearest residence	0.110	(-)
Slope	0.038	(-)
Hare-Rabbit	0.150	(+)
Distance to the nearest road	0.061	(-)
NDVI	0.083	(+)

**Table 9:** Beta estimates and standard errors for covariates included in all equally plausible models for mara occupancy.

		PSI (OCCUPANCY)							
MODEL		Densroad	Cow-Horse	Goat-Sheep	Distres	Slope	HR	Distroad	NDVI
psi(densroad),p(.)	Beta	-0.543	0	0	0	0	0	0	0
	SE	0.285	0	0	0	0	0	0	0
psi(densroad,cow-horse),p(.)	Beta	-0.549	2.059	0	0	0	0	0	0
	SE	0.2901	1.628	0	0	0	0	0	0
psi(densroad,goat-sheep),p(.)	Beta	-0.541	0	1.328	0	0	0	0	0
	SE	0.2945	0	1.05	0	0	0	0	0
psi(densroad,distres),p(.)	Beta	-0.559	0	0	-0.318	0	0	0	0
	SE	0.290	0	0	0.277	0	0	0	0
psi(densroad,cow-horse,goat-sheep),p(.)	Beta	-0.546	1.981	1.293	0	0	0	0	0
	SE	0.300	1.611	1.058	0	0	0	0	0
psi(slope,goat-sheep,hr),p(.)	Beta	0	0	1.761	0	-0.423	4.048	0	0
	SE	0	0	1.049	0	0.285	2.496	0	0
psi(densroad,distroad),p(.)	Beta	-0.553	0	0	0	0	0	-0.152	0
	SE	0.284	0	0	0	0	0	0.253	0
psi(hr),p(.)	Beta	0	0	0	0	0	3.843	0	0
	SE	0	0	0	0	0	2.705	0	0
psi(densroad,distroad,goat-sheep),p(.)	Beta	-0.552	0	1.349	0	0	0	-0.159	0
	SE	0.295	0	1.059	0	0	0	0.254	0
psi(distres,hr),p(.)	Beta	0	0	0	-0.37	0	4.499	0	0
	SE	0	0	0	0.276	0	2.875	0	0
psi(ndvi,hr),p(.)	Beta	0	0	0	0	0	3.767	0	0.331
	SE	0	0	0	0	0	2.666	0	0.242
psi(ndvi,cow-horse),p(.)	Beta	0	2.470	0	0	0	0	0	0.385
	SE	0	1.691	0	0	0	0	0	0.245
psi(ndvi,distres,hr),p(.)	Beta	0	0	0	-0.371	0	4.324	0	0.336
	SE	0	0	0	0.276	0	2.773	0	0.245

elev = elevation,densroad = road density, distres = distance to the nearest residence, distroad = distance to the nearest road, cow-horse = proportion of transect with signs of cows or horses, goat-sheep = proportion of transect with signs of goats or sheep, hr = proportion of transect with signs of introduced rabbits and/or hares.

For guanacos, the detection probability model with lowest AIC contained the observer as a covariate (Table 3). The principal observer had a higher detection probability than the secondary observers. Probability of detection of signs was high, ranging from 0.73 to 0.95 at the different sites under the different models (Table 10). Five models were equally plausible. In terms of covariates related to habitat, guanacos were more likely to be found in higher, steeper areas. Livestock, both goats/sheep and cows/horses, was negatively associated with guanacos. Among the covariates related to hunting, guanacos were more likely to be found where there were fewer roads (Tables 11 and 12). Goats/sheep was the most important covariate, with almost twice the weight of slope, the next covariate with the next highest importance weight (Table 11). Probability of occupancy of different sites as estimated under the different models ranged from near zero to 0.84, with an overall weighted average CV of 58% (Table 10).

**Table 10:** Guanaco model with lowest AIC and all models within a delta AIC of <2.

							PSI				P			
							Estimate		SE		Estimate		SE	
MODEL	AIC	Delta AIC	AIC weight	Model Likelihood	Number of parameters	-2. Log Likelihood	MIN	MAX	MIN	MAX	MIN	MAX	MIN	MAX
psi(slope,elev,goat-sheep),p(obs)	151.5	0	0.185	1	6	139.5	0.0001	0.772	0.0003	0.225	0.728	0.944	0.031	0.117
psi(slope,goat-sheep),p(obs)	151.69	0.19	0.168	0.909	5	141.69	0.0003	0.841	0.001	0.172	0.732	0.945	0.031	0.115
psi(cow-horse,goat-sheep),p(obs)	152.43	0.93	0.116	0.628	5	142.43	0.001	0.664	0.002	0.129	0.729	0.945	0.031	0.116
psi(elev,goat-sheep),p(obs)	152.59	1.09	0.107	0.58	5	142.59	0.0001	0.677	0.0003	0.130	0.729	0.944	0.031	0.116
psi(densroad,cow-horse,goat-sheep),p(obs)	152.73	1.23	0.1	0.541	6	140.73	0.0004	0.738	0.001	0.143	0.726	0.945	0.031	0.117

Minimum (MIN) and maximum (MAX) occupancy (PSI) and detectability (P) estimates and minimum and maximum standard errors for Psi and P for individual sites for each model. elev = elevation,densroad = road density, cow-horse = proportion of transect with signs of cows or horses, goat-sheep = proportion of transect with signs of goats or sheep, obs = observer.

**Table 11:** Importance weight and direction of relationship of each covariate for guanaco occupancy.

VARIABLE	IMPORTANCE WEIGHT	RELATIONSHIP
Goat-Sheep	0.676	(-)
Slope	0.353	(+)
Elevation	0.292	(+)
Cow-Horse	0.216	(-)
Road density	0.1	(-)

**Table 12:** Beta estimates and standard errors for covariates included in all equally plausible models for guanaco occupancy.

			PSI (OCCUPANCY)				P (DETECTABILITY)
MODEL		Goat-Sheep	Slope	Elev	Cow-Horse	Densroad	Obs2
psi(slope,elev,goat-sheep),p(obs)	Beta	-11.030	0.425	0.362	X	X	-1.849
	SE	3.645	0.254	0.248	X	X	0.817
psi(slope,goat-sheep),p(obs)	Beta	-9.871	0.480	X	X	X	-1.832
	SE	3.241	0.258	X	X	X	0.820
psi(cow-horse,goat-sheep),p(obs)	Beta	-8.678	X	X	-2.830	X	-1.848
	SE	2.966	X	X	1.641	X	0.817
psi(elev,goat-sheep),p(obs)	Beta	-10.611	X	0.413	X	X	-1.843
	SE	3.545	X	0.241	X	X	0.812
psi(densroad,cow-horse,goat-sheep),p(obs)	Beta	-9.334	X	X	-2.840	-0.310	-1.862
	SE	3.124	X	X	1.653	0.246	0.818

elev = elevation,densroad = road density, cow-horse = proportion of transect with signs of cows or horses, goat-sheep = proportion of transect with signs of goats or sheep, obs2 = observer other than the principal observer (relative to principal observer).

When importance weights for covariates were summed across species, the covariate with the greatest weight was goat and sheep density (Table 13). This overall importance weight was negative even though the relationship with maras was positive, due to the heavy negative weights for guanacos and rheas. Road density had the next greatest importance, as it had a negative weight for all three species. Although distance to the nearest road was not associated with guanaco presence, this was the covariate with the next greatest weight, due to its negative association with rheas and maras. The overall importance weights of cow-horse and distance to the nearest rural residence were also negative.

**Table 13:** Overall importance weights for covariates related to human activities.

VARIABLE	IMPORTANCE WEIGHT	RELATIONSHIP
Goat-Sheep	0.8903	(-)
Road density	0.5727	(-)
Distance to road	0.3959	(-)
Cow-Horse	0.1205	(-)
Distance to residence	0.0663	(-)

## Discussion

Importance weights obtained for covariates related to different threats allow us to establish hypotheses to guide conservation actions for adaptive intervention within this landscape, a method that could be used for similar conservation planning in other areas. The analysis is not meant to enable strong conclusions about explanatory power of the covariates [6], nor are the specific results meant to be extrapolated to other landscapes. The most important factor associated with distribution of the suite of species in the landscape was goat and sheep density. This suggests that interventions that reduce the impact of livestock would have the greatest impacts on the conservation of these species. However, the research does not identify the mechanism through which goats and sheep are negatively associated with lesser rheas and guanacos, and positively with maras, so our initial conservation actions must be based on hypotheses about these mechanisms, supplemented by prior research and other information from the landscape.

Possible mechanisms for the strong negative relationship between goat and sheep density and lesser rheas and guanacos include direct and indirect competition, habitat degradation resulting from heavier grazing in areas with more goats and sheep, and persecution by or greater presence of goat and sheep herders in areas used more heavily by their livestock. In studies in other parts of Patagonia, lesser rheas did not appear to be negatively affected by high numbers of sheep nor greatly affected by overgrazing, did not have a high dietary overlap with sheep, and intense hunting and egg harvest appeared to have a stronger effect than overgrazing on their density and reproductive success [21], [33]. Thus we hypothesize that for rheas, the mechanism for the negative relationship with sheep and goats in our area could be persecution by or greater presence of herders in areas with more sheep and goats. Alternatively, goats are much more common than sheep in this area, and we cannot rule out a negative impact of greater competition with goats compared to sheep. Also, top predators such as pumas *Puma concolor* and culpeos *Lycalopex culpaeus*, are abundant in the landscape, with frequent attacks on livestock [34] so we cannot rule out an “apparent competition” effect of high numbers of goats supplementing predators, which in turn limit less abundant populations of lesser rheas [13].

For guanacos, the mechanism for the negative relationship with livestock is most likely direct competition. The fact that guanacos were found in the drier areas makes habitat degradation an unlikely mechanism. Other studies have found a strong negative relationship between guanacos and sheep [23],[35], and goat [20] density, and have provided evidence that the mechanism is direct competition for forage [14], [24], [36]. However, persecution by herders and ranchers and their dogs is common (pers. obs.) and may also contribute to the negative relationship found in this landscape.

The mara is much more of a habitat specialist than the other two species, and its positive association with livestock may be because of a preference for more open habitat, due to a strategy for escaping predation based on early detection and fast flight to the safety of a den. Maras have higher reproductive success in more open areas [37], [38], and more open areas are temporarily covered with annual grasses in spring, resulting in increased food resources at this critical time of year [39]. Areas in this study with more livestock may be more open from heavy grazing and trampling. High densities of sheep and goats may also increase resource richness for maras via fertilization of vegetation with their dung [40]. In spite of the possibility of facilitation of maras by livestock, the strong negative association with rheas and guanacos indicates that it is important to work with rural residents to reduce persecution and to find ways to reduce direct competition, by decreasing livestock densities, making changes in livestock management, or improving range condition to increase availability of forage.

The high summed importance weight for road density indicates that the next most important factor to address for all three species in this landscape is illegal hunting from roads, which is done mostly by hunters from towns, cities and oil camps. In southern Patagonia, guanaco occurrence increased with distance from cities and oil camps, the common sources of poachers [23]. In a previous study within our study area, density of roads, including old oil exploration trails, was the most important factor affecting guanaco density in and around a protected area [17]. Habitat associations with more open spaces may bring maras into greater contact with urban hunters. Closing unused oil trails that provide access to urban hunters with vehicles and increasing ranger patrols are the principal interventions to address this type of hunting, and our results suggest that this could have a positive impact on the entire suite of large herbivores.

The strong negative importance weight for road density seems to contradict our results showing that maras and lesser rheas were more likely to be found closer to roads, as we had conceptualized both road density and distance to roads as indicators of pressure of hunting by poachers from vehicles. Both species were more likely to be found nearer to main roads, even though their negative associations with road density suggest they are negatively impacted by hunting from roads. Maras may use roads and trails as corridors between different portions of their home ranges [39], and both species may be attracted to forage alongside roads where livestock is excluded, in spite of the hunting risk. Rheas were also more likely to be found at greater distances from rural residences, and rhea meat and eggs are coveted foods for rural people in the area [41]. Rheas are difficult to hunt from a vehicle, and the traditional method of hunting is with a “boleadora”, a rock attached to rope that is swung by a hunter on horseback to entangle the rhea’s legs. Although this hunting is illegal, it is widespread in this landscape (pers. obs.). We hypothesize that the greater probability of finding rheas near roads may be in part because rural hunters are less likely to carry out this conspicuous form of illegal hunting near public roads where they might be spotted by rangers or other passersby. Therefore, rheas, unlike guanacos, which are easier to hunt from a vehicle, may find refuge from hunting by rural residents near public roads, similar to the sheltering effect of roads from predators that has been found for herbivores in North America [42], [43]. It is more difficult for rangers to monitor and control hunting from horseback than hunting from vehicles. For this type of hunting working directly with people to reduce their motives for hunting may be required.

Our previous studies in the area showed that direct estimation (based on sightings) of rheas and maras is difficult because detectability in some habitat types is very low [26]. Population estimations of lesser rheas based on sign transects with a calibrated index are greater than those based on direct counts, indicating that many animals go undetected in direct counts, as is the case for many species [8]. Robustness of estimates based on signs may be increased by including easily-collected covariates that affected detection probability in this study, such as the observer and the time of day. Guanacos are easily observed directly, and their abundance can be monitored by vehicular transects. However, the random walking transects in this study based on signs perhaps reduce bias caused by vehicular transects limited to areas with roads. In addition, monitoring occupancy throughout the landscape based on signs is quicker and can give a better indication of the level of connectivity, particularly in areas of low guanaco density, between the few abundant populations. Nevertheless, our walking transects failed to detect guanacos in some areas where we know they are present, indicating that our overall estimate of area occupied by guanacos is biased low. We suggest that even for species for which line transects based on sightings are feasible, such data may be complemented at a landscape level by occupancy data at random sites based on signs.

Although detection probabilities were high for lesser rheas and guanacos, the low detection probabilities for the mara indicate that the design may not be appropriate for monitoring this species [44]. Random linear transects may fail to detect the species due to its patchy distribution and habitat use. The cell size of the grid may also be large for this species that is smaller-bodied and a central place forager [40]. When considering sign-based occupancy studies such as this one for monitoring multiple species, adaptations may be required to the study design to provide robust estimates for different species. In general, detection probabilities of at least 0.15 are necessary to obtain reliable estimates of occupancy [44].

With just a few months of fieldwork, this study provided key guidelines for planning and prioritizing conservation actions for the three main target species in the landscape. We consider our conclusions to be working hypotheses that may be altered as wildlife responses to interventions are monitored. The method provided new insights into the landscape-level effects on the guanaco, and showed how the lesser rhea, may still suffer from hunting pressure even if poaching by urban hunters is controlled. For all species the methodology generated important information for planning conservation interventions and designing landscape-level monitoring of the effectiveness of those interventions. This sign-based method may be adapted for any large landscape where a rapid, objective means for prioritizing conservation actions on multiple species is needed and data on the relative importance of different human activities affecting these species are scarce.
